# Overexpression of EphB6 and EphrinB2 controls soma spacing of cortical neurons in a mutual inhibitory way

**DOI:** 10.1038/s41419-023-05825-w

**Published:** 2023-05-06

**Authors:** Chun-Hui He, Ning-Ning Song, Pin-Xi Xie, Yu-Bing Wang, Jia-Yin Chen, Ying Huang, Ling Hu, Zhao Li, Jun-Hui Su, Xiao-Qing Zhang, Lei Zhang, Yu-Qiang Ding

**Affiliations:** 1 Key Laboratory of Arrhythmias, Ministry of Education of China, East Hospital, and Department of Anatomy and Neurobiology, Tongji University School of Medicine, Shanghai, 200092 China; 2 Department of Laboratory Animal Science, Fudan University, Shanghai, 200032 China; 3 State Key Laboratory of Medical Neurobiology and MOE Frontiers Center for Brain Science, Institutes of Brain Science, Fudan University, Shanghai, 200032 China; 4 Shanghai Yangzhi Rehabilitation Hospital (Shanghai Sunshine Rehabilitation Center) and Department of Anatomy, Histology and Embryology, Tongji University School of Medicine, Shanghai, 200092 China; 5 Clinical Center for Brain and Spinal Cord Research, Tongji University, Shanghai, 200092 China; 6 Department of Anesthesiology, Xiangya Hospital Central South University, Changsha, Hunan 410008 China; 7 Shanghai Tongji Hospital, Tongji University School of Medicine, Shanghai, 200092 China

**Keywords:** Neuronal development, Brain

## Abstract

To establish functional circuitry, neurons settle down in a particular spatial domain by spacing their cell bodies, which requires proper positioning of the soma and establishing of a zone with unique connections. Deficits in this process are implicated in neurodevelopmental diseases. In this study, we examined the function of EphB6 in the development of cerebral cortex. Overexpression of EphB6 via *in utero* electroporation results in clumping of cortical neurons, while reducing its expression has no effect. In addition, overexpression of EphrinB2, a ligand of EphB6, also induces soma clumping in the cortex. Unexpectedly, the soma clumping phenotypes disappear when both of them are overexpressed in cortical neurons. The mutual inhibitory effect of EphB6/ EphrinB2 on preventing soma clumping is likely to be achieved via interaction of their specific domains. Thus, our results reveal a combinational role of EphrinB2/EphB6 overexpression in controlling soma spacing in cortical development.

## Introduction

Development of the cerebral cortex including soma placing, dendritic arborization, axonal targeting, and synaptic connectivity requires sensitive and precise regulatory mechanisms [1, 2]. During cortical development, different types of neurons form a highly ordered six-layer cortical structure in an “inside to outside” pattern through the continuous process of neural stem cell proliferation, neuronal differentiation and migration. After migration to final destination, immature neurons evenly fill a mosaic spacing and uniform dendritic coverage with neighboring homotypic neurons and adopt a mature identity characterized by connectivity and gene expression [3–5]. Disruptions of any process, including the specification of cell body position contribute to the etiology of many neurodevelopmental disorders [6]. Thus, deciphering the molecular basis responsible for the regulation of soma spacing is essential for understanding the maturation of brain functions and defining pathogenic mechanisms.

Neurons develop their appropriate soma spacing during development usually requires a self-avoidance or homotypic avoidance mechanism [7]. Self-avoidance ensures that neurons of the homotypic space themselves nonrandomly relative to each other and fill a particular spatial domain, as is the case in the vertebrate retina [8, 9]. One strategy to allow self-avoidance is to use diverse molecules expressed in a given type of neurons to mediate this recognition event. In mice, Dscam-expressing retina neurons are “indifferent” to one another but when mutant for it, instead, excessively adhere with themselves [10, 11]. However, the molecular mechanisms underlying the process remain elusive in mammalian brain.

Erythropoietin-producing hepatocellular (Eph) receptor Tyr kinases together with its membrane-tethered ligands, the Ephrins, elicit short-distance cell-cell signaling and thus involved in various signal transduction processes in the organogenesis as well as in the pathogenies of cancers [12, 13]. Within the nervous system, almost all the Eph receptors and Ephrins are expressed in the developing and adult nervous system [14, 15] and are implicated in neuron migration, axonal outgrowth and pathfinding, axon fasciculation, and distribution of neocortical neurons [15–25]. Our previous studies showed that EphA7 functions downstream of Satb2 in regulating soma placing and dendritic self-avoidance of cortical pyramidal neurons [26, 27].

Unlike other Eph receptors, EphB6 is a kinase-deficient Eph receptor and highly conserved [28, 29]. A recent study showed that although kinase-dead, EphB6 remains a strategically active component of Eph receptor signaling [30]. It is expressed in a wide variety of tissues and cell types, including the brain [29, 31], and EphB6 has a biphasic function in cell adhesion and migration in vitro [32]. EphrinB2 has been reported to be a candidate ligand for EphB6 [33]. Recently, EphB6 was shown to be colocalized with vesicular glutamate transporter 1 and vesicular GABA transporter, suggesting that EphB6 is present in both excitatory and inhibitory synapses [34]. In addition, genomic studies have suggested that EphB6 is a candidate autistic spectrum disorder (ASD)-associated gene [35–37], and autism-like behavior is observed in EphB6-deficient mice [38]. Most importantly, transcriptome analyses have shown that EPHB6 is upregulated in patients with ASD [39]. However, biological functions of this receptor in brain development are largely unknown.

In this study, we investigated the role of EphB6 in cortical development by gain-of-function and loss-of-function approaches with the help of *in utero* electroporation (IUE) in mouse. We found that knockdown of EphB6 has no effect, but its overexpression results in soma clumping, in which all domains of EphB6 are required. In addition, we showed that overexpression of EphrinB2, the ligand of EphB6, also induces soma clumping. Notably, the deficit of soma placing is no longer existed when both are overexpressed. Furthermore, we propose a *cis*-attenuation mechanism between EphB6 and EphrinB2 via interaction of their specific domains. Therefore, our results indicate a mutual inhibitory mechanism is applied in overexpression of EphB6/EphrinB2-controled soma spacing during cortical development and have implications for neurodevelopmental disorders.

## Materials and methods

### Experimental animals

Wild-type ICR mice at the age of 2–3 months were maintained on a 12-h light/dark cycle in the animal facility of Tongji University. For staging of embryos, mid-day of the vaginal plug identified was calculated as embryonic day 0.5 (E0.5). The day of birth was considered postnatal day 0 (P0). No randomization was used for allocation of animals to experimental groups. No blinding was done for group allocation during the experiments or when assessing the outcome. All animal protocols used in this study were reviewed and approved by the Animal Committee of Tongji University School of Medicine, Shanghai, China.

### DNA constructs

For gene overexpression constructs, specific primers containing Flag coding sequence were used to amplify desired DNA fragments from mouse cortical cDNA library. The PCR products were cloned into the XhoI/XmaI sites of pCAGGS vector. For preparation of constructs expressing truncated forms of EphB6 and EphrinB2, splicing by overlap extension (SOE) PCR was carried out by using gene specific and mutagenic specific primers, followed by cloning in pCAGGS vector. The sequence of the EphB6-short hairpin RNA (shRNA) construct was targeted against nucleotides 555-573 (5′-GGTTCTAGGGTCATCAGTT-3′) of mouse EphB6 mRNA (NM_001146351). The complementary oligonucleotides were annealed and inserted into the pSUPER-EGFP vector (pSUPER for short), which itself alone was used as a control for EphB6-shRNA. The shRNA plasmid was constructed as described previously [26, 40].

### HEK293T cell culture, transfection and Western blot

Cell cultures and Western blots were performed as described previously [40, 41]. Human embryonic kidney (HEK) 293T cells obtained from CCTCC were recently tested for mycoplasma contamination but not authenticated. Briefly, HEK293T cells were maintained in DMEM (Gibco) supplemented with 10% fetal bovine serum (Hyclone). Cells of about 70%-80% confluence were transfected using lipofectamine 8000 (Beyotime) according to the manufacturer’s instructions. If shRNA was used, the ratio of shRNA to expression constructs is 1:1. Forty-eight hours later, cells were lysed in ice-cold radioimmunoprecipitation (RIPA) buffer containing protease inhibitor cocktail (Thermo Scientific). Samples were then loaded on sodium dodecyl sulfate-polyacrylamide gel electrophoresis (SDS-PAGE). After transfer to membrane, the samples were then probed with rabbit anti-FLAG antibody (1:1000; #14793, CST) or rabbit anti-GAPDH antibody (1:1000; LF206, Epizyme), developed with species-specific horseradish peroxidase (HRP)-conjugated secondary antibodies (1:2000; LF102, Epizyme) and visualized with enhanced chemiluminescence (Epizyme). For all Western blots, three cultures for each group were used.

### *In utero* electroporation (IUE)

IUE was performed according to previous procedures [42]. Briefly, pregnant mice at E14.5 or E15.5 were anesthetized with sodium pentobarbital (40 mg/kg), and embryos in the uterus were carefully exposed and placed on humidified gauze pads. A mixture of plasmid (1 μg/μl) with pCAGGS-EGFP (1 μg/μl) was injected into the lateral ventricles of the embryonic brain. pCAGGS-EGFP was injected into different embryos that were served as control. After the injection, five square electric pulses (33 V) were delivered through the uterus at 1-s interval, with the forcep-type electrodes connected to the electroporator (BTX ECM 830; Harvard Instrument) while the uterus was kept wet with warm saline. The uterus was then placed back into the abdominal cavity, where the abdominal wall and skin were sutured with a surgical needle and thread. The pregnant mouse was warmed in an incubator until it regains consciousness. The embryos or pups were allowed to continue developing normally for the time indicated.

### Immunohistochemistry and in situ hybridization (ISH)

For immunostaining of HEK293T cells, cells were cultured on coverslips before transfection. Forty-eight hours after transfection with pCAGGS-EphB6, pCAGGS-EphrinB2 or their truncated forms, cells were fixed with 4% paraformaldehyde (PFA) for 10 min. Cells were then blocked in 10% bovine serum albumin/PBS and incubated with rabbit anti-Flag (1:1,000; #14793, CST) antibody at 4°C overnight. Cells were incubated with Cy3-conjugated rabbit secondary antibodies (1:500; 016160084, Jackson ImmunoResearch) and Hoechst 33258 (Invitrogen) for counterstaining.

Embryos at different stages and pups at different postnatal stages were deeply anaesthetized by sodium pentobarbital (40 mg/kg body weight) and perfused with PBS (pH7.4), followed by 4% PFA. Then the brains were dissected out, post-fixed for 24 h at 4°C and cryoprotected with 30% sucrose in PBS for another 24 h at room temperature. Coronal sections at 12 μm (embryos) or 25 μm (pups) thickness were prepared using a cryostat (CM1950, Leica) and then subjected to immunohistochemistry or ISH immediately.

For immunohistochemistry, antigen retrieval using citrate buffer was performed prior to the blocking step. The following primary antibodies were used: goat anti-GFP (1:2000; NB100-1770, Novus Biologicals), rabbit anti-GFP (1:1000; A11122, Invitrogen), rabbit anti-Cleaved Caspase3 (Asp175) (1:300; #9661, CST), goat anti-Nestin (1:500; sc-21248, Santa Cruz), rabbit anti-PH3 (1:1000; #9701, CST) and rabbit anti-Sox2 (1:500; ab97959, abcam) antibodies. Primary antibodies were detected with species-specific Alexa Fluor 488- (1:500; A-11055 or A-21206, Invitrogen) or Cy3-conjugated (1:1000; 016160084, Jackson ImmunoResearch) antibodies for 3 h at room temperature. For BrdU labeling, one pulse of BrdU (100 mg/kg body weight, i.p.) was administered to pregnant mice with E15.5 embryos. Twenty-four hours later, mice were killed, and brains were sectioned as mentioned above. Before incubation with BrdU (1:500; OBT0030CX, Bio-Rad), brain slices were treated with 0.01 M sodium citrate at 95 °C for 5 min, 2 N HCl for 20 min at 37 °C, 0.1 M borate buffer (pH 8.5) for 10 min at room temperature.

For ISH, antisense digoxigenin-labeled RNA probes for EphB6 and EphrinB2 were synthesized and ISH was performed as described previously [43]. These two probes were constructed according to the descriptions on the website of the Allen Brain Atlas. Double labeling of ISH and GFP immunostaining was performed as described previously [41].

### Image acquiring and analysis

Images were acquired using Nikon Eclipse 80i, Nikon A1R or Leica SP8 microscopes. Images were processed using Adobe Photoshop CC 2018. GFP fluorescence line intensity plots along the medial-lateral axis of the cortex were quantified using a previously described method [23, 44, 45]. Briefly, regions for quantification were delimited from medial to lateral in the cortex, straightened with ImageJ software and the plot profiles of the fluorescent intensity was made as fluorescence gray values (*y*) against the normalized tangential distance (*x*). For cell counts of Cleaved Caspase3/GFP, BrdU/GFP, PH3/GFP, Sox2/GFP colocalization, all double-positive and GFP-positive cells in the same field were counted and the ratio of double-positive cells to GFP-positive cells was calculated. Data were collected from more than three different embryos or pups.

### Statistical analysis

Statistical analysis was performed using the GraphPad Prism software, version 8.3.0. All data were tested for normal distribution and homogeneity of variance and all data met the assumptions of the tests. Comparison was performed using unpaired Student’s *t*-test. All data were presented as mean ± SEM. Statistical significance is indicated as *p < 0.05 and **p < 0.01. n.s., *p* > 0.05. The value of n and *p* for each graph was stated in figure legends. No statistical methods were used for sample size estimate.

## Results

### EphB6 is dynamically expressed in developing cerebral cortex

**Fig. 1:**
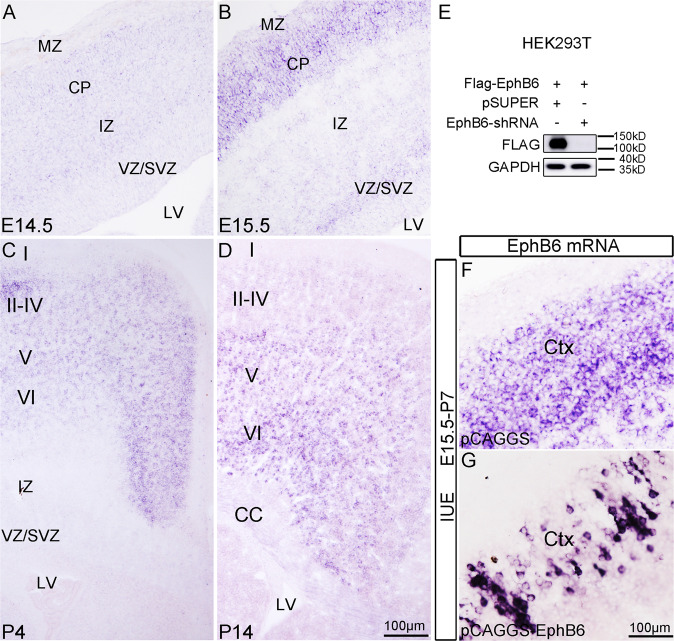
EphB6 is dynamically expressed in developing cerebral cortex. **A**–**D** Expression of EphB6 revealed by ISH in the cortex at different developmental stages. **E** Western blot shows that transfection of EphB6-shRNA into HEK293T cells greatly reduces expression of exogenous EphB6. **F**, **G** ISH signals for EphB6 in control (**F**) and EphB6-electroporated cortex (**G**). VZ ventricular zone, SVZ subventricular zone, IZ intermediate zone, CP cortical plate, MZ marginal zone, CC corpus callosum. I–VI, layers of cortex. Scale bars, 100 μm in (**A**–**D**) and (**F**, **G**).

In order to elucidate possible functions of EphB6 in cortical development, we examined its expression in the cerebral cortex at different stages using ISH. EphB6 mRNA was detected with a very low level, if any, in the cortical plate with no ISH signals in the ventricular zone (VZ) or subventricular zone (SVZ) at E14.5 (Fig. 1A). The expression of EphB6 became evident in the cortical plate at E15.5, and weak ISH signals were observed in the SVZ (Fig. 1B). At P4, EphB6 transcripts were present throughout the cerebral cortex with high level in cortical layers II-IV relative to that in layer V–VI, but were absent in VZ/SVZ or IZ (Fig. 1C). At P14, EphB6 mRNA was expressed predominantly in layers V–VI, and was not observed in SVZ (Fig. 1D). The dynamic expression in the cortex suggests potential roles of EphB6 in cortical development.

### Overexpression of EphB6 results in clumping of neuronal cell bodies

A previous report has shown that EphB6 is involved in cell adhesion and migration in vitro [32]. In this study, we carried out loss-of-function and gain-of-function approaches to examine whether EphB6 is implicated in cortical development.

We generated shRNA expression vector that specifically targets mouse EphB6 (hereafter referred to as EphB6-shRNA). Western blots were used to examine the efficiency of EphB6-shRNA, and it showed that Flag-tagged exogenous EphB6 protein was dramatically reduced when EphB6-shRNA was co-transfected in HEK293T cells relative to those transfected with empty pSUPER vector (Fig. 1E and S6 for full-length uncropped picture). We also generated a Flag-tagged EphB6-overexpressing construct (pCAGGS-EphB6) by inserting EphB6 coding sequence (CDS, GenBank: NM_001146351.2) into pCAGGS vector, and its expression of EphB6 was confirmed in HEK293T cells, and cortical tissues with IUE of this vector (Fig. 1E, G). Noted that the electroporated neurons contained more intense ISH signals for EphB6 mRNA compared with those of endogenous transcripts (Fig. 1F, G).

**Fig. 2:**
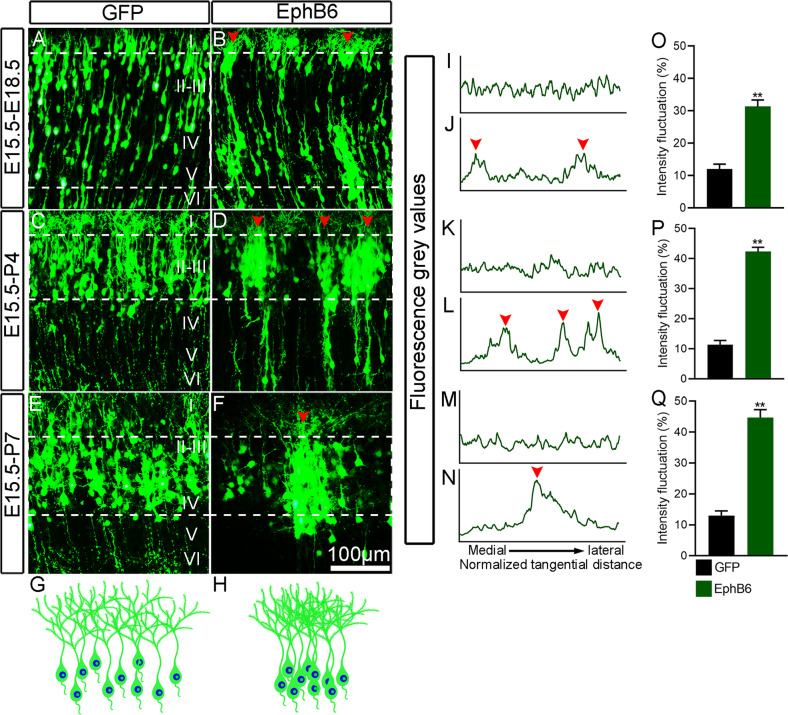
EphB6 overexpression results in clumping of cell bodies. **A**–**F** Distribution of GFP-labelled neurons in the cortex. EphB6 overexpression plasmid was delivered by IUE at E15.5 and examined at E18.5, P4 and P7, respectively. Arrowheads indicate the clusters of neurons. **G**, **H** Schematic diagram showing the distribution of GFP-labeled neurons in control and EphB6-electroporated cortex. **I**–**N** Fluorescence grey values were quantified from the images in (**A**–**F**) from left to right (medial to lateral in the cortex) as delineated by dashed lines. Plot profiles indicate the clustering of neurons with overexpression of EphB6 (arrowheads in **B**, **D**, **F** and **J**, **L**, **N**). The *x*-axis represents the normalized tangential distance from medial to lateral and arrowheads indicate the peak levels of the fluorescence intensity. **O**–**Q** Quantification of the fluorescence intensity fluctuations by measuring the intensity between minima and maxima and the normalized distance (dashed line in **A**–**F**). Bar graphs are plotted as mean ± SEM, **p < 0.01, Student’s *t*-test, *n* = 3 brains. Scale bar, 100 μm in (**A**–**F**).

We next electroporated EphB6-shRNA or pCAGGS-EphB6 plasmid into dorsolateral VZ of the forebrain at E15.5 to manipulate EphB6 expression in neural progenitors that give rise to layer II/III neurons. pCAGGS-EGFP was simultaneously delivered to trace electroporated neurons. We analyzed the distribution of GFP^**+**^ cells in the cortex at E18.5 and P4. Knockdown of EphB6 did not obviously affect the distribution of neurons in the cortical plate at E18.5, and the cortical neurons were evenly distributed in layers II/III of the cortex at P4 with no difference compared with control brain (Figure S1A–F). To analyze the distribution of the GFP^+^ neurons, we calculated the normalized intensity profile of the fluorescence gray values in the cortical plate (Figure S1G–J), which revealed similar fluctuations in the density of control and EphB6-shRNA neurons (Figure S1K, L). In contrast, overexpression of EphB6 led to a striking alteration of the tangential distribution of cortical neurons, as shown by the presence of soma clumping in cortical plate at E18.5, and in the cortex at P4 and P7 (Fig. 2A–H). The normalized GFP intensity profile in the cortical plate (Fig. 2I–N) revealed substantial fluctuations in the density of neurons with overexpression of EphB6 (Fig. 2O–Q). Thus, the overexpression of EphB6 leads to soma clumping of cortical neurons.

To examine whether overexpression of EphB6 affects cortical apical progenitors, we performed IUE of pCAGGS-EphB6 at E14.5, administered BrdU at E15.5 and sacrificed the mice at E16.5. The expression of proliferation marker BrdU, mitotic cell marker PH3 and apical progenitor marker Sox2 were then checked. More GFP-labeled cells were BrdU^+^, PH3^+^ or Sox2^+^, respectively (Figure S2B–D). These results indicate EphB6-overexpression maintains the apical progenitors in the progenitor state instead of differentiation. This deficit seems to be transient because most transfected neurons managed to migrate into superficial layers in the cortex postnatally (data not shown). Further, to investigate whether EphB6-overexpression induces cell apoptosis/death and affects radial glial scaffold, double immunostaining for GFP and Cleaved Caspase3 was performed in E18.5 brain with IUE at E15.5, and there were no significant differences in the ratio of Cleaved Caspase3^+^/GFP^+^ cells to the total GFP^+^ cells or the total number of Cleaved Caspase3-postive cells between the two groups (Figure S3A–D). In addition, Nestin immunostaining showed that in the EphB6 overexpressed cortex, Nestin^+^ radial glial fibers were oriented straightly toward the pial surface, similar to the controls (Figure S3E–J). Taken together, these results showed that overexpression of EphB6 neither induces apoptosis/cell death nor affects the architecture of radial glial fibers.

### Overexpression of EphB6 affects soma spacing in a cell-autonomous way

**Fig. 3:**
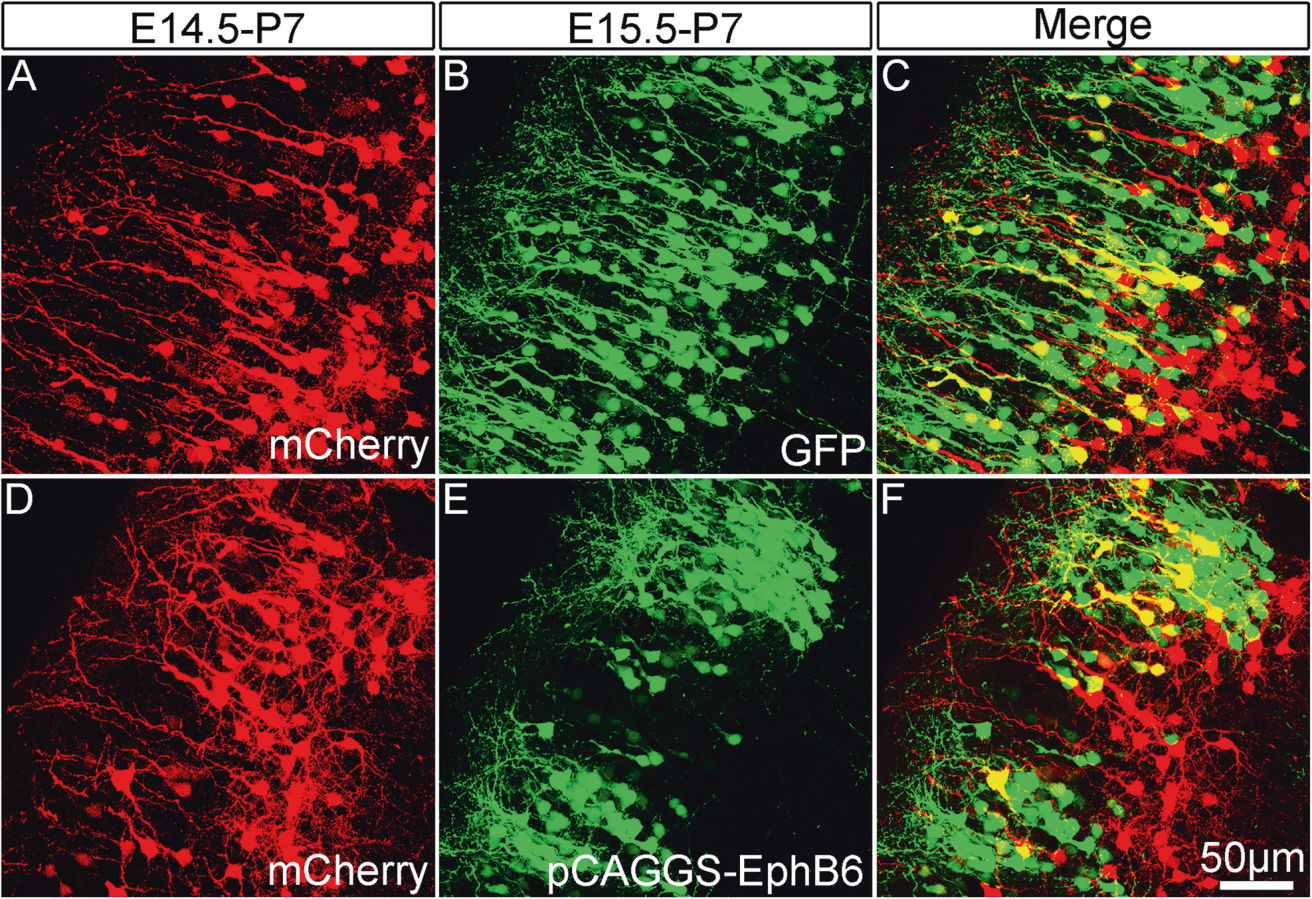
Sequential *in utero* electroporation reveals a cell-autonomous effect of EphB6-induced soma clumping. **A**–**C** Distribution of cortical neurons electroporated with pCAGGS-mCherry at E14.5 and those electroporated with pCAGGS-GFP on E15.5 in the same cortex at P7. Note that mCherry-labeled neurons are located deeper in the cortex than GFP-labeled neurons. **D**–**F** Neurons transfected with pCAGGS-mCherry at E14.5 exhibit even cell bodies spacing, but those with pCAGGS-EphB6 at E15.5 display clumping phenotype at P7. Scale bar, 50 μm in (**A**–**F**).

Although Eph usually exerts biological effects in a non-cell autonomous manner, some observations have shown that the Eph receptors can signal independently of ephrin ligands, i.e., functioning in a cell-autonomous way [46–48]. To examine whether EphB6 controls the soma spacing in a cell-autonomous way, we performed sequential IUE to separately label two pools of cortical neurons with overexpression of EphB6 in one of the pools. Individual embryos were electroporated with pCAGGS-mCherry to label wild-type neurons at E14.5 and transfected again to overexpress pCAGGS-EphB6 at E15.5. As shown in Fig. 3, mCherry-labeled wild-type neurons were uniformly distributed in the superficial layers without the clumping of neuronal cell bodies (Fig. 3A–C), and cortical neurons with overexpression of pCAGGS-EphB6 showed the phenotype as mentioned above (Fig. 3D–F). These results indicated that the overexpression of EphB6 shows cell-autonomous effects on soma spacing.

### All domains of EphB6 are required to induce deficits in soma spacing

**Fig. 4:**
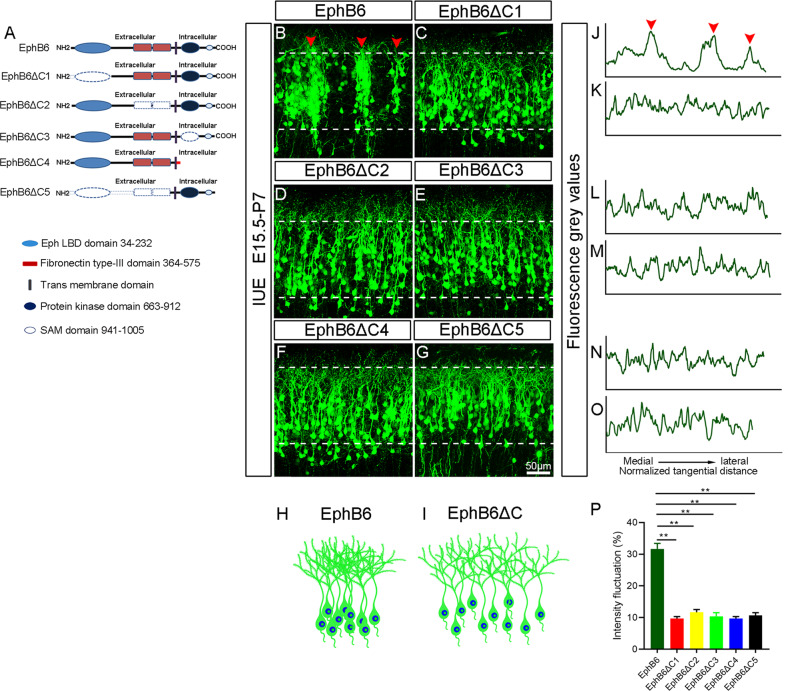
All domains in EphB6 are required for inducing deficits of neuronal tangential distribution. **A** Diagrams of full-length EphB6 and five truncated forms. The dotted box indicates that this domain has been truncated. **B**–**G** Representative images of cortical coronal sections electroporated with the indicated plasmids at E15.5 and examined at P7. Arrowheads indicate the clusters of neurons. **H**, **I** Schematic diagram showing the distribution of GFP-labeled neurons in EphB6 overexpression and EphB6ΔC-electroporated cortices. **J**–**O** Plot profiles indicate the fluorescence intensity in the cortical regions marked with dashed lines in (**B**–**G**), respectively. The x-axis represents the normalized tangential distance from medial to lateral and arrowheads indicate the peak levels of the fluorescence gray values. **P** Quantification of the fluorescence intensity fluctuations in those dashed lines portions of the cortex. Bar graphs are plotted as mean ± SEM, **p < 0.01, Student’s *t*-test, *n* = 3 brains. Scale bar, 50 μm in (**B**–**G**).

**Fig. 5:**
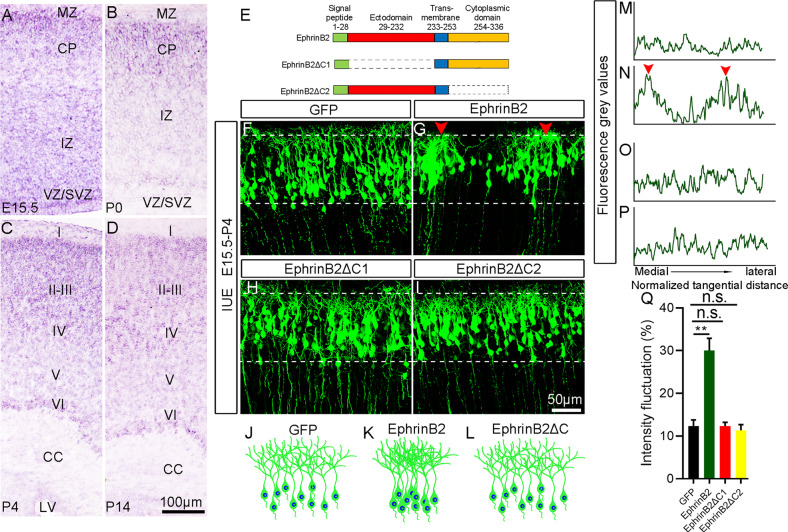
EphrinB2 is also involved in cortical soma spacing. **A**–**D** Expression of EphrinB2 revealed by ISH at different developmental stages. VZ ventricular zone, SVZ subventricular zone, IZ intermediate zone, CP cortical plate, MZ marginal zone, CC corpus callosum. I–VI, cortical layers. **E** Schematic representation of the structures of full length EphrinB2 and its truncated forms. The dotted box indicates that this domain is truncated. **F**–**I** Representative images of cortical neurons electroporated with the indicated plasmids at E15.5 and examined at P4. Arrowheads indicate the clusters of neurons. **J**–**L** Schematic diagram showing the distribution of GFP-labeled neurons in the cortex electroporated with control, EphrinB2 overexpression and truncated forms of EphrinB2. **M**–**P** Plot profiles indicate the fluorescence intensity in the areas indicated by dashed lines in panels (**F**–**I**), respectively. The x-axis represents the normalized tangential distance from medial to lateral and arrowheads indicate the peak levels of the fluorescence intensity. **Q** Quantification of the intensity fluctuations in the cortical regions labeled with dashed lines in (**F**–**I**). Bar graphs are plotted as mean ± SEM, n.s., *p* > 0.05, **p < 0.01, Student’s *t*-test, *n* = 3 brains. Scale bars, 100 μm in (**A**–**D**) and 50 μm in (**F**–**I**).

EphB6 is a transmembrane protein and composed of several domains [30], we next moved to examine which domain(s) is necessary for regulating soma positioning. As shown in Figs. 4A, 5 truncated EphB6-Flag expression plasmids (EphB6ΔC1 lacking Ephrin binding domain, EphB6ΔC2 without Fibronectin type III domain, EphB6ΔC3 without tyrosine kinase-dead domain, EphB6ΔC4 missing most of the intracellular domain and EphB6ΔC5 missing entire extracellular domain) were prepared. The expression of full-length EphB6 and truncated EphB6 in HEK293T cells and on the cell membrane was confirmed by Western blots and immunostaining (Fig. S4A, C-H and S6 for full-length uncropped picture). Then we electroporated them individually into the embryos at E15.5 and examined them at P7. We found that the GFP^+^ cells transfected with the truncated forms exhibited a homogeneous distribution in the cortex, unlike those cells in EphB6-overexpressing brains showing soma clumping (Fig. 4B–I). The normalized fluorescence intensity plot profile (Fig. 4J–O) and fluctuation of density from medial to lateral cortical plates (Fig. 4P) in brains transfected with the truncated forms were significantly different from EphB6-overexpressing brains. Thus, all domains of EphB6 are required in affecting soma placing of cortical neurons.

### EphrinB2 is also involved in cortical soma spacing

EphrinB2 is a candidate ligand of EphB6 [33], and this promoted us to explore if EphrinB2 is also implicated in regulating soma placing of cortical neurons,. ISH data showed that EphrinB2 was expressed throughout the CP, IZ and VZ/SVZ at E15.5 (Fig. 5A). At P0, EphrinB2 expression level was low in the IZ and VZ/SVZ, while high expression was observed in the CP (Fig. 5B). At P4 and P14, EphrinB2 was expressed in all layers of cerebral cortex with a similar density, but almost absent in SVZ (Fig. 5C, D).

We next performed IUE of full-length EphrinB2 at E15.5 and analyzed the distribution of GFP^**+**^ cells at P4. Like the case of overexpression of EphB6, the soma clumping phenotype was observed, and the fluorescence intensity of GFP^**+**^ neurons fluctuated significantly (Fig. 5F, G, J, K, M, N, Q). EphrinB2 was shown to induce signalling cascades involving receptor-independent activities [46], so next we constructed two truncated forms of EphrinB2 expression plasmids: a mutant form of EphrinB2 lacking the EphB receptor binding site (EphrinB2ΔC1) and a truncated form lacking the entire intracellular domain (EphrinB2ΔC2) (Fig. 5E); their expressions were also confirmed by Western blots and immunostaining in HEK293T cells (Fig. S4B, I-K and S6 for full-length uncropped picture). We overexpressed these two truncated forms at E15.5, examination of P4 brains revealed a homogeneous distribution of electroporated neurons in the cortex (Fig. 5H–Q). Thus, overexpression of EphrinB2, a ligand of EphB6 receptor also results in soma clumping in developing cerebral cortex, and like the case of EphB6 all domains of EphrinB2 are required in inducing this phenotype. In addition, unlike EphB6, overexpression of EphrinB2 in apical progenitors had no effects on their proliferation (Fig. S2E–G).

### Overexpression of EphB6 and EphrinB2 regulates soma spacing in a mutual inhibitory way

In general, Eph receptors and their ligand Ephrins are reciprocally compartmentalized during embryogenesis. However, Eph-A receptors and Ephrin-A ligands are also expressed simultaneously in an overlapping manner [49, 50], and Eph receptors and its ligands interact *trans* on opposite membranes and *cis* on the same membrane [51]. We first examined the expression of EphrinB2 in the neighboring cells when EphB6 was overexpressed, and found no changes in the expression of EphrinB2 (Fig. S5A, B). Similarly, we failed to detect any changes of EphB6 expression in neighboring neurons after EphrinB2 overexpression (Fig. S5C, D). These results were also consistent with our data in sequential IUE (Fig. 3).

**Fig. 6:**
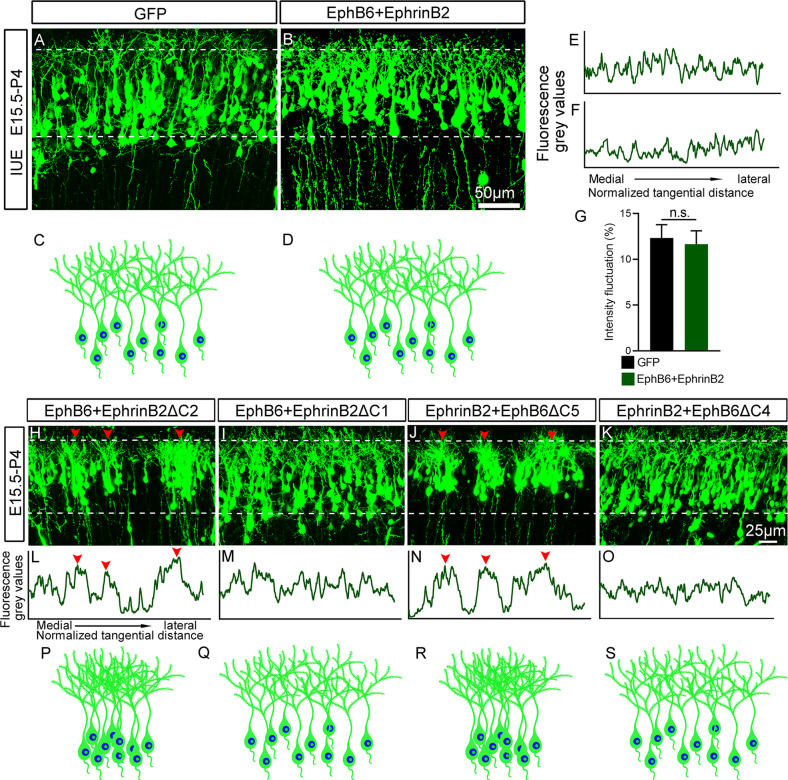
Overexpression of EphB6 and EphrinB2 regulates soma spacing in a mutual inhibitory way. **A**, **B** P4 cortex electroporated with control (**A**) and both EphB6 and EphrinB2 expression plasmid at E15.5 (**B**). Note that the neurons with overexpression of both EphB6 and EphrinB2 were uniformly distributed. **C**, **D** Schematic diagram showing the distribution of GFP-labeled neurons in control and Ephb6 and EphrinB2 co-transfected cortices. **E**, **F** Plot profiles indicate the fluorescence gray values in the areas labeled by dashed lines in panels (**A**, **B**). **G** Quantification of the intensity fluctuations in the cortical regions labeled with dashed lines in (**A**, **B**). Bar graphs are plotted as mean ± SEM, n.s., *p* > 0.05, Student’s *t*-test, *n* = 3 brains. **H**–**K** Mouse embryos were electroporated *in utero* at E15.5 with indicated combinations of two plasmids and analyzed at P4. Arrowheads indicate the clusters of neurons. **L**–**O** Fluorescence grey values were quantified from the images in (**H**–**K**), respectively, from left to right (medial to lateral in the cortex) as delineated by dashed lines. Plot profiles indicate the clustered neurons in the cortex (arrowheads in **H**, **L** and **J**, **N**). **P**–**S** Represents the distribution diagram of GFP-labeled neurons in (**H**–**K**). Scale bars, 50 μm in (**A**, **B**) and 25 μm in (**H**–**K**).

**Fig. 7:**
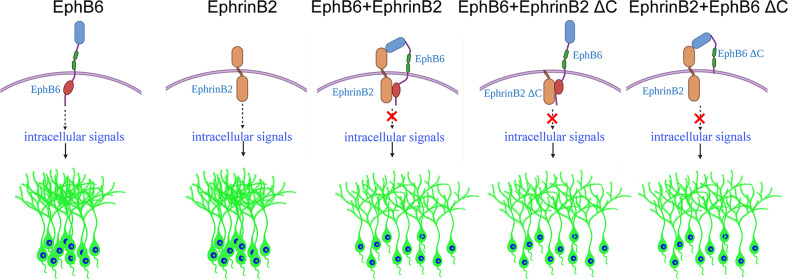
Proposed working model for EphB6/EphrinB2 gain of function in cortical soma spacing. Overexpression of EphB6 receptor or EphrinB2 results in soma clumping of cortical neurons, whereas simultaneous overexpression of both eliminates the clumping phenotype. The interaction of extracellular domain of EphB6 with EphrinB2, and EphB6 with intracellular domain of EphrinB2 are the key components that may restore intracellular signaling pathways that are responsible for soma spacing impaired by overexpression EphB6 or EphrinB2.

Having found overexpression of either EphB6 or EphrinB2 leads to soma clumping, we were curious about what happens when both were co-overexpressed in the same neurons. To this end, we overexpressed both full-length EphB6 and EphrinB2 at E15.5 and examined the distribution of cortical neurons at P4. Suprisingly, cortical neurons with overexpession of both displayed a homogeneous distribution in the cortex, comparable to that in control brains (Fig. 6A-G). Considering the findigns that Ephs and Ephrins interact on the surface of the same cell (*cis*), which attenuates their signalings [13, 51–54], we hypothesized that EphB6 receptor and its ligand EphrinB2 may interact directly with *in cis* through their specific domains, and thus weakening their signaling (Fig. 7).

To obtain more evidence supporting the hypothesis, we co-overexpressed full-length of EphB6 or EphrinB2 with a truncated form of the other, that is, the EphrinB2ΔC1, EphrinB2ΔC2 and EphB6ΔC4 as mentioned above, as well as the EphB6ΔC5 without entire extracellular domain. As shown in Fig. 6H–S, the soma clumping phenotype was eliminated in the combinations of full-length EphB6 with EphrinB2ΔC1, and full-length EphrinB2 with EphB6ΔC4, but not other combinations. These results indicate that the extracellular domain of EphB6 and intracellular domain of EphrinB2 are the key elements in mediating the mutual inhibitory effects of EphB6/EphrinB2 overexpression in preventing the soma clumping (Fig. 7).

## Discussion

In this study, we examined the roles of EphB6 and its ligand EphrinB2 in cortical development and found that overexpression of either EphB6 or EphrinB2 impaired the tangential cell distribution of cortical pyramidal neurons as shown by the presence of soma clumping. Interestingly, the soma clumping phenotypes disappeared when both were co-expressed in the cortical neurons, thus revealing a novel mechanism underlying overexpression of EphB/EphrinB-regulated soma allocation of cortical neurons during the morphogenesis of cerebral cortex.

The major approach we used in this study is *in utero* electroporation (IUE), which directly targets the cortical apical progenitors in VZ and indirectly their progeny neurons migrating to the cortex later. Thus, it is important to explore the consequences after overexpression of EphB6 or EphrinB2 in apical progenitors in the first place. As shown in Fig. S2, with overexpression of EphB6, more GFP positive cells were also positive for BrdU (a proliferation marker), PH3 (a mitotic cell marker), and Sox2 (an apical progenitor marker), indicating EphB6 expression tend to maintain the cells in the progenitor state. While overexpression of EphrinB2 did not show any effects on apical progenitors. However, most of neurons with overexpression of EphB6 managed to migrate to the superficial layers postnatally, just similar to the control cells, indicating that this increase in proliferation might be a transient deficit during development.

The cerebral cortex is organized along two main axes: tangential and radial [55]. During cortical development, postmitotic neurons migrate from the proliferative zone along the radial glial scaffold and constitute ontogenetic columns to reach their final laminar destinations [1, 19, 56]. Recently, Eph/Ephrin has been shown to participate in controlling the lateral dispersion (i.e., tangential migration) of cortical pyramidal neurons [19, 23]. Specifically, overexpression of EphrinB1 in postmitotic neurons induces the formation of the compact clusters in developing cerebral cortex [19]. Our previous study showed that Satb2 is required for soma spacing and dendritic self-avoidance in developing cortex [26]. In searching downstream genes mediating Satb2-controlled soma spacing, we found that EphA7 expression is elevated in Satb2-deficient neurons, and overexpressing EphA7 also induces soma clumping phenotype in the cortex while reducing its expression in Satb2-deficient neurons prevents soma clumping [27]. In this study, we demonstrated additional Eph/Ephrin member, EphB6 and EphrinB2 is also implicated in tangential distribution of cortical neurons as shown by the data that soma clumping was present in cortical neurons with overexpression of EphB6 or EphrinB2. However, it should be noted EphB6 may function in a Satb2-independent way as EphB6 expression is reduced rather than increased in Satb2-deficient cortical neurons [57]. Collectively, the neuronal soma clumping can be observed after overexpression of several members in Eph:Ephrin family, including EphA7, EphB6 and EphrinB2. We speculate that gain of function of these receptors/ligands enhances intracellular signaling which promotes cell adhesion. As EphB6 and Ephrinb2 are potential receptor/ligand, they may form *trans-* or *cis-* interaction and *cis*- interaction has been shown to inhibit signaling [58]. EphB6 and EphA7 are both receptors and belong to two different binding specificity classes (class A and B) [59], and thus theoretically they cannot make any types of interaction. In addition, overexpression of EphB6 in cortical neurons had no effect on the expression of EphrinB2 (Figure S5) or soma spacing (Fig. 3) in neighboring untransfected neurons, it is unlikely that this cell-autonomous effect of EphB6 would involve EphA7 function.

In many areas of the brain, neurons are distributed in organized spatial arrangements. Previous studies have shown abnormal distribution of neurons in the brain of autistic patients, which is manifested as patches of disorganization [60]. The abnormal distribution of neurons caused by overexpression of EphB6 is similar to this phenotype. In addition, transcriptomic sequencing found that EPHB6 is a risk gene for autism, and that EphB6 expression was elevated in whole blood genomic profiling tests of autistic patients [39]. Therefore, EPHB6 is very likely to be implicated in the pathogenesis of ASD. It will be interesting in the future to examine the influence of EphB6 and EphrinB2-induced abnormal distribution on neuronal activity and animal behavior.

EphB6 is a transmembrane protein with an extracellular domain, which is involved in ligand recognition, and a cysteine-rich cytoplasmic domain, which are involved in mediating protein-protein interactions and receptor dimerization. Our results showed that these two domains are involved in perturbation of tangential distribution of cortical neurons in EphB6-overexpressed cortex, because deletion either of them did not affect the soma spacing. EphrinB2 is proposed to be a ligand for EphB6 [33], and it thus is not surprising that overexpression of EphrinB2 also disturbs the tangential distribution of cortical neurons.

On the other hand, it is unexpected that the soma clumping phenotype disappears when EphB6 and EphrinB2 were co-overexpressed. As mentioned early, Eph signaling is attenuated by a *cis*-interactions between Eph receptors and Ephrins on the same cell [51, 61]. For example, *cis* interaction between EphA3 and EphrinA5 abolishes the induction of tyrosine phosphorylation of EphA3 [53]. Ephrin also mediate *cis*-attenuation of EphA signaling in spinal cord motor neurons, which is essential for spinal motor axon guidance [52], and even distribution Cajal-Retzius cells in the superficial cortex is a consequence of Eph/Ephrins-mediated contact repulsion [55]. The explanation for these effects may be that binding sites for *trans*-interactions are masked by the co-expression of Eph and Ephrin in the same cell [51] or possibly by inhibiting the formation of Eph clusters [52]. In our study, when EphB6 or EphrinB2 overexpressed, it may regulate the recruitment of various effector proteins by intracellular signaling. These effector proteins then influence cytoskeleton organization, leading to soma clumping. But when EphB6 and EphrinB2 are co-expressed simultaneously, they may interact directly in *cis* via their specific domains or co-expressed EphrinB2 can fine-tune the responsiveness of EphB6, blocking/attenuating abnormal downstream intracellular signaling and associated alteration of cytoskeleton induced by the overexpression (Fig. 7). Further studies are needed to explore the downstream effector molecules and signaling pathways.

In summary, EphB6 and its ligand EphrinB2 are involved in the soma positioning in the developing cerebral cortex, via a mutual inhibitory fashion. Soma spacing is an essential prerequisite of establishing proper neuronal connections and also brain functions. Abnormal arrangement of neural cell bodies is observed in neurodevelopmental diseases such as autism, for which EPHB6 is a risk gene. Our data provide further evidence for the link between neuronal disorganization and these diseases.

## Supplementary information

Supplementary figures and legends

Reproducibility checklist

## Data Availability

All data generated or analyzed during this study are included in this published article and its Supplementary Information files.

## References

[1] Greig LC, Woodworth MB, Galazo MJ, Padmanabhan H, Macklis JD (2013). Molecular logic of neocortical projection neuron specification, development and diversity. Nat Rev Neurosci.

[2] Kwan KY, Sestan N, Anton ES (2012). Transcriptional co-regulation of neuronal migration and laminar identity in the neocortex. Development.

[3] Rakic P (2009). Evolution of the neocortex: a perspective from developmental biology. Nat Rev Neurosci.

[4] Molyneaux BJ, Arlotta P, Menezes JR, Macklis JD (2007). Neuronal subtype specification in the cerebral cortex. Nat Rev Neurosci.

[5] Popovitchenko T, Rasin M-R (2017). Transcriptional and post-transcriptional mechanisms of the development of neocortical lamination. Front Neuroanat.

[6] Romero DM, Bahi-Buisson N, Francis F (2018). Genetics and mechanisms leading to human cortical malformations. Semin Cell Dev Biol.

[7] Wässle H, Boycott BB (1991). Functional architecture of the mammalian retina. Physiol Rev.

[8] Cook JE, Chalupa LM (2000). Retinal mosaics: new insights into an old concept. Trends Neurosci.

[9] Fuerst PG, Burgess RW (2009). Adhesion molecules in establishing retinal circuitry. Curr Opin Neurobiol.

[10] Fuerst PG, Koizumi A, Masland RH, Burgess RW (2008). Neurite arborization and mosaic spacing in the mouse retina require DSCAM. Nature.

[11] Fuerst PG, Bruce F, Tian M, Wei W, Elstrott J, Feller MB (2009). DSCAM and DSCAML1 function in self-avoidance in multiple cell types in the developing mouse retina. Neuron.

[12] Pasquale EB (2010). Eph receptors and ephrins in cancer: bidirectional signalling and beyond. Nat Rev Cancer.

[13] Kania A, Klein R (2016). Mechanisms of ephrin–Eph signalling in development, physiology and disease. Nat Rev Mol Cell Biol.

[14] Tuzi NL, Gullick WJ (1994). eph, the largest known family of putative growth factor receptors. Br J Cancer.

[15] Yun ME, Johnson RR, Antic A, Donoghue MJ (2003). EphA family gene expression in the developing mouse neocortex: regional patterns reveal intrinsic programs and extrinsic influence. J Comp Neurol.

[16] Ferri RT, Levitt P (1993). Cerebral cortical progenitors are fated to produce region-specific neuronal populations. Cereb Cortex.

[17] Torres R, Firestein BL, Dong H, Staudinger J, Olson EN, Huganir RL (1998). PDZ proteins bind, cluster, and synaptically colocalize with Eph receptors and their ephrin ligands. Neuron.

[18] Conover JC, Doetsch F, Garcia-Verdugo JM, Gale NW, Yancopoulos GD, Alvarez-Buylla A (2000). Disruption of Eph/ephrin signaling affects migration and proliferation in the adult subventricular zone. Nat Neurosci.

[19] Dimidschstein J, Passante L, Dufour A, van den Ameele J, Tiberi L, Hrechdakian T (2013). Ephrin-B1 controls the columnar distribution of cortical pyramidal neurons by restricting their tangential migration. Neuron.

[20] Gatto G, Morales D, Kania A, Klein R (2014). EphA4 receptor shedding regulates spinal motor axon guidance. Curr Biol.

[21] Peuckert C, Wacker E, Rapus J, Levitt P, Bolz J (2008). Adaptive changes in gene expression patterns in the somatosensory cortex after deletion of ephrinA5. Mol Cell Neurosci.

[22] Yokoyama N, Romero MI, Cowan CA, Galvan P, Helmbacher F, Charnay P (2001). Forward signaling mediated by ephrin-B3 prevents contralateral corticospinal axons from recrossing the spinal cord midline. Neuron.

[23] Torii M, Hashimoto-Torii K, Levitt P, Rakic P (2009). Integration of neuronal clones in the radial cortical columns by EphA and ephrin-A signalling. Nature.

[24] Tai AX, Kromer LF (2014). Corticofugal projections from medial primary somatosensory cortex avoid EphA7-expressing neurons in striatum and thalamus. Neuroscience.

[25] Son AI, Hashimoto-Torii K, Rakic P, Levitt P, Torii M (2016). EphA4 has distinct functionality from EphA7 in the corticothalamic system during mouse brain development. J Comp Neurol.

[26] Zhang L, Song NN, Chen JY, Huang Y, Li H, Ding YQ (2012). Satb2 is required for dendritic arborization and soma spacing in mouse cerebral cortex. Cereb Cortex.

[27] He CH, Zhang L, Song NN, Mei WY, Chen JY, Hu L (2022). Satb2 regulates EphA7 to control soma spacing and self-avoidance of cortical pyramidal neurons. Cereb Cortex.

[28] Gurniak CB, Berg LJ (1996). A new member of the Eph family of receptors that lacks protein tyrosine kinase activity. Oncogene.

[29] Matsuoka H, Iwata N, Ito M, Shimoyama M, Nagata A, Chihara K (1997). Expression of a kinase-defective Eph-like receptor in the normal human brain. Biochem Biophys Res Commun.

[30] Strozen TG, Sharpe JC, Harris ED, Uppalapati M, Toosi BM (2021). The EphB6 receptor: kinase-dead but very much alive. Int J Mol Sci.

[31] Shimoyama M, Matsuoka H, Tamekane A, Ito M, Iwata N, Inoue R (2000). T-cell-specific expression of kinase-defective Eph-family receptor protein, EphB6 in normal as well as transformed hematopoietic cells. Growth Factors.

[32] Matsuoka H, Obama H, Kelly ML, Matsui T, Nakamoto M (2005). Biphasic functions of the kinase-defective Ephb6 receptor in cell adhesion and migration. J Biol Chem.

[33] Munthe E, Rian E, Holien T, Rasmussen A, Levy FO, Aasheim H (2000). Ephrin-B2 is a candidate ligand for the Eph receptor, EphB6. FEBS Lett.

[34] Bartolini G, Sanchez-Alcaniz JA, Osorio C, Valiente M, Garcia-Frigola C, Marin O (2017). Neuregulin 3 Mediates Cortical Plate Invasion and Laminar Allocation of GABAergic Interneurons. Cell Rep..

[35] Butler MG, Rafi SK, Manzardo AM (2015). High-resolution chromosome ideogram representation of currently recognized genes for autism spectrum disorders. Int J Mol Sci.

[36] O’Roak BJ, Deriziotis P, Lee C, Vives L, Schwartz JJ, Girirajan S (2011). Exome sequencing in sporadic autism spectrum disorders identifies severe de novo mutations. Nat Genet.

[37] Iossifov I, O’Roak BJ, Sanders SJ, Ronemus M, Krumm N, Levy D (2014). The contribution of de novo coding mutations to autism spectrum disorder. Nature.

[38] Li Y, Luo ZY, Hu YY, Bi YW, Yang JM, Zou WJ (2020). The gut microbiota regulates autism-like behavior by mediating vitamin B6 homeostasis in EphB6-deficient mice. Microbiome.

[39] Gregg JP, Lit L, Baron CA, Hertz-Picciotto I, Walker W, Davis RA (2008). Gene expression changes in children with autism. Genomics.

[40] Zhang L, Huang Y, Chen JY, Ding YQ, Song NN (2015). DSCAM and DSCAML1 regulate the radial migration and callosal projection in developing cerebral cortex. Brain Res.

[41] Zhang L, Song NN, Zhang Q, Mei WY, He CH, Ma P (2020). Satb2 is required for the regionalization of retrosplenial cortex. Cell Death Differ.

[42] Ding YQ, Kim JY, Xu YS, Rao Y, Chen ZF (2005). Ventral migration of early-born neurons requires Dcc and is essential for the projections of primary afferents in the spinal cord. Development.

[43] Ding YQ, Marklund U, Yuan W, Yin J, Wegman L, Ericson J (2003). Lmx1b is essential for the development of serotonergic neurons. Nat Neurosci.

[44] Bonnin A, Torii M, Wang L, Rakic P, Levitt P (2007). Serotonin modulates the response of embryonic thalamocortical axons to netrin-1. Nat Neurosci.

[45] Farias GG, Guardia CM, Britt DJ, Guo X, Bonifacino JS (2015). Sorting of dendritic and axonal vesicles at the pre-axonal exclusion zone. Cell Rep..

[46] Bochenek ML, Dickinson S, Astin JW, Adams RH, Nobes CD (2010). Ephrin-B2 regulates endothelial cell morphology and motility independently of Eph-receptor binding. J Cell Sci.

[47] Foo SS, Turner CJ, Adams S, Compagni A, Aubyn D, Kogata N (2006). Ephrin-B2 controls cell motility and adhesion during blood-vessel-wall assembly. Cell.

[48] Defourny J, Audouard C, Davy A, Thiry M (2021). Efnb2 haploinsufficiency induces early gap junction plaque disassembly and endocytosis in the cochlea. Brain Res Bull.

[49] Flanagan JG, Vanderhaeghen P (1998). The ephrins and Eph receptors in neural development. Annu Rev Neurosci.

[50] Marquardt T, Shirasaki R, Ghosh S, Andrews SE, Carter N, Hunter T (2005). Coexpressed EphA receptors and ephrin-A ligands mediate opposing actions on growth cone navigation from distinct membrane domains. Cell.

[51] Yin Y, Yamashita Y, Noda H, Okafuji T, Go MJ, Tanaka H (2004). EphA receptor tyrosine kinases interact with co-expressed ephrin-A ligands in cis. Neurosci Res.

[52] Kao TJ, Kania A (2011). Ephrin-mediated cis-attenuation of Eph receptor signaling is essential for spinal motor axon guidance. Neuron.

[53] Carvalho RF, Beutler M, Marler KJ, Knoll B, Becker-Barroso E, Heintzmann R (2006). Silencing of EphA3 through a cis interaction with ephrinA5. Nat Neurosci.

[54] Mao Y-T & Dalva MB. EphB-ephrinB cis interaction regulates filopodial movement. 2019. https://jdc.jefferson.edu/neuroscienceposters/1.

[55] Villar-Cerviño V, Molano-Mazón M, Catchpole T, Valdeolmillos M, Henkemeyer M, Martínez LM (2013). Contact repulsion controls the dispersion and final distribution of Cajal-Retzius cells. Neuron.

[56] Tan SS, Breen S (1993). Radial mosaicism and tangential cell dispersion both contribute to mouse neocortical development. Nature.

[57] Alcamo EA, Chirivella L, Dautzenberg M, Dobreva G, Farinas I, Grosschedl R (2008). Satb2 regulates callosal projection neuron identity in the developing cerebral cortex. Neuron.

[58] Arvanitis D, Davy A (2008). Eph/ephrin signaling: networks. Genes Dev.

[59] Wilkinson DG (2001). Multiple roles of EPH receptors and ephrins in neural development. Nat Rev Neurosci.

[60] Stoner R, Chow ML, Boyle MP, Sunkin SM, Mouton PR, Roy S (2014). Patches of disorganization in the neocortex of children with autism. N. Engl J Med.

[61] Bohme B, VandenBos T, Cerretti DP, Park LS, Holtrich U, Rubsamen-Waigmann H (1996). Cell-cell adhesion mediated by binding of membrane-anchored ligand LERK-2 to the EPH-related receptor human embryonal kinase 2 promotes tyrosine kinase activity. J Biol Chem.

