# Supplementary material for: Overexpression of EphB6 and EphrinB2 controls soma spacing of cortical neurons in a mutual inhibitory way
Source: Cell Death Dis. 2023 May 6;14(5):309. doi: 10.1038/s41419-023-05825-w (PMC10164173; doi:10.1038/s41419-023-05825-w)
Supplement: Supplementary file 1 — Supplementary figures and legends [file 41419_2023_5825_MOESM1_ESM.docx]

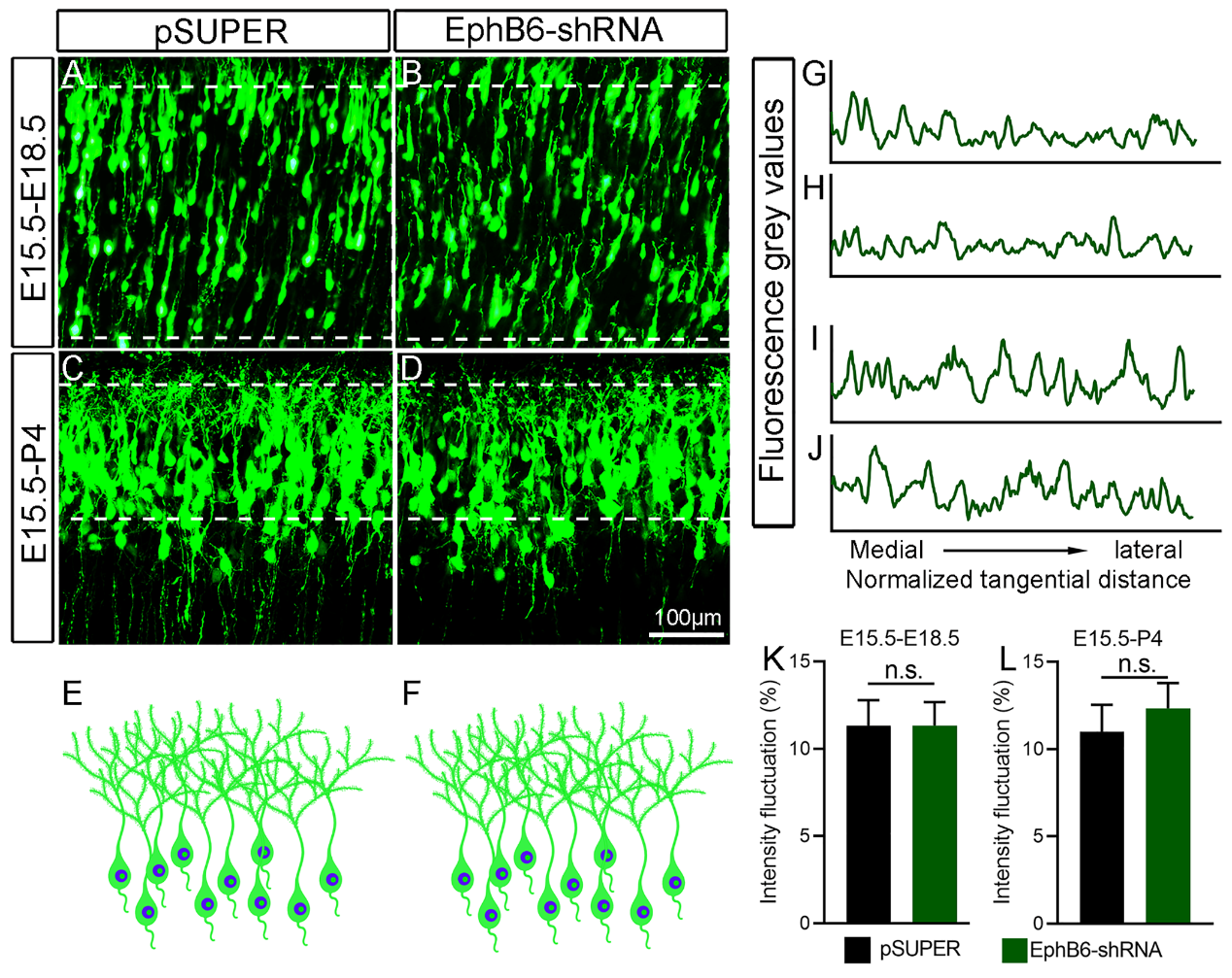


**Fig. S1 Knockdown of EphB6 does not significantly affect tangential distribution of cortical neurons.**

(A-D) Representative images of coronal sections of mouse brains electroporated with control or EphB6 shRNA plasmid at E15.5 and examined at E18.5 or P4 respectively. (E-F) Schematic diagram showing the distribution of GFP-labeled cells in control and Ephb6-shRNA transferred cortex.

(G-J) Plot profiles indicate the fluorescence intensity in the areas labeled by dashed lines in panels (A-D). The x-axis represents the normalized tangential distance from medial to lateral.

(K-L) Quantification of the intensity fluctuations in the cortical regions labeled with dashed lines in (A-D). Bar graphs are plotted as mean ± SEM, n.s., nonsignificant, *p* > 0.05, Student’s *t* test, n = 3 brains. Scale bar,100 μm in (A-D).


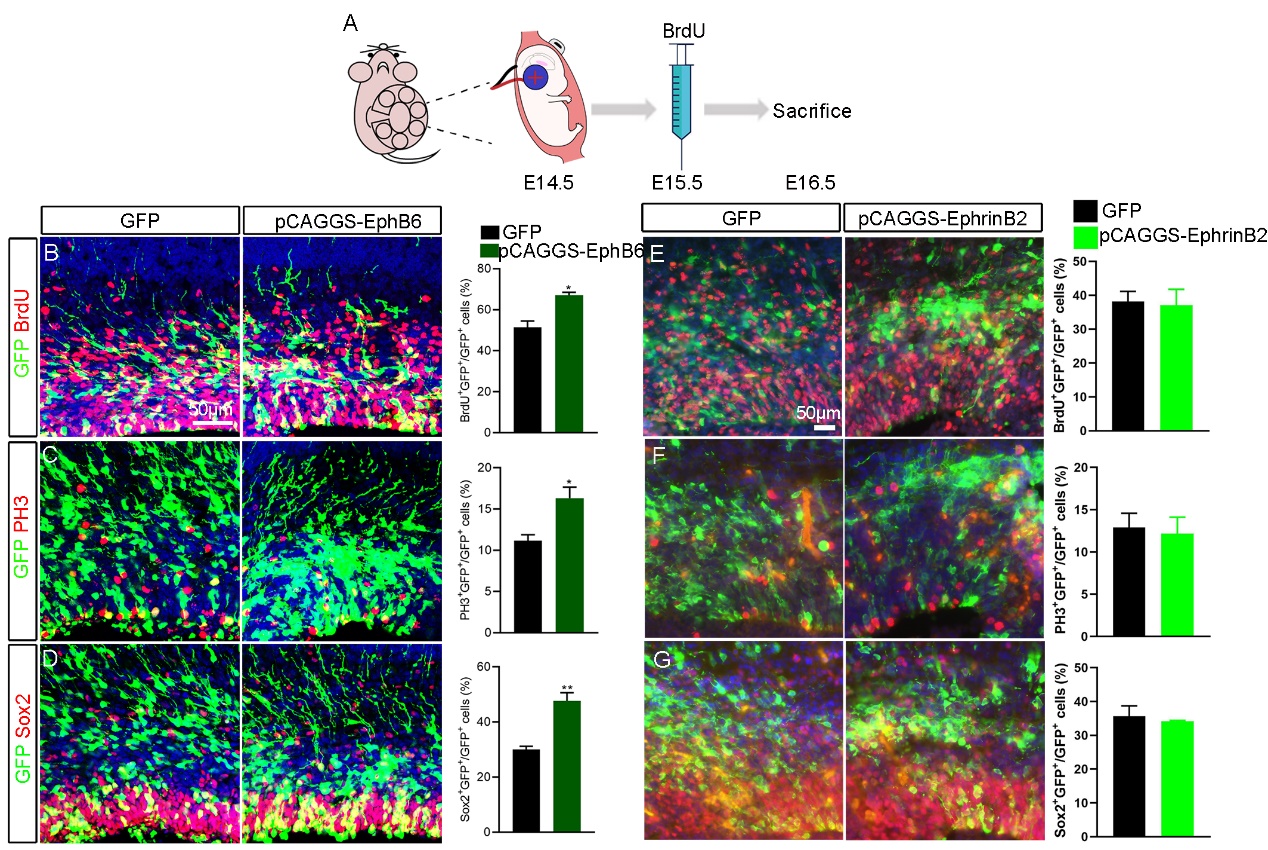
**Fig. S2 Overexpression of EphB6 increases the proliferation of apical progenitors, but overexpression of EphrinB2 does not.**

(A) Schematic diagram of IUE and following BrdU injection and mouse sacrifice at designated time point.

(B-D) More GFP^+^ cells are labeled with BrdU (proliferation marker), PH3 (mitotic cell marker) or Sox2 (apical progenitor marker) by immunostaining in EphB6-overexpressing mouse brains, as shown by a higher ratio of BrdU^+^/GFP^+^, PH3^+^/GFP^+^ or Sox2^+^/GFP^+^ to total GFP^+^ cells.

(E-G) The ratio of BrdU^+^/GFP^+^, PH3^+^/GFP^+^ and Sox2^+^/GFP^+^ to total GFP^+^ cells were comparable between GFP control and EphrinB2-overexpressing mouse brains.

Data are shown as mean ± SEM. *p<0.05, **p<0.01, Student’s *t* test, n = 3 brains. Scale bars, 50 μm in B-D and 50 μm in E-G.


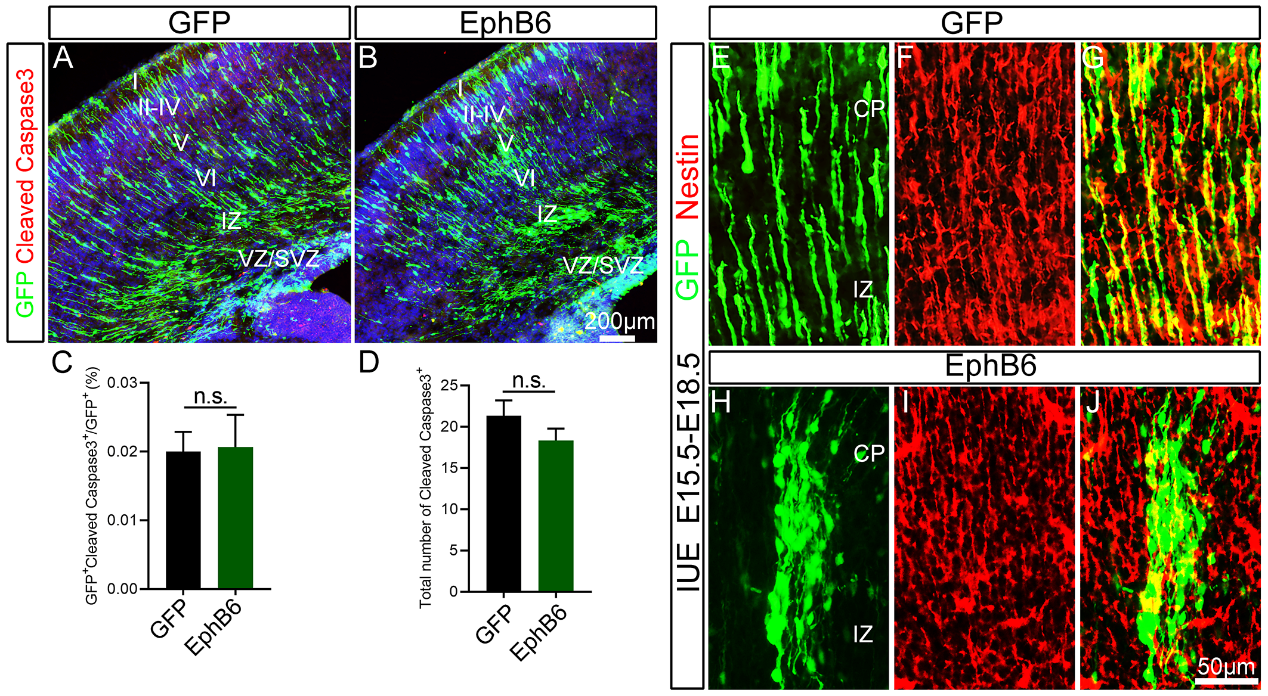


**Fig. S3 Overexpression of EphB6 does not induce apoptotis/cell death and not affect the architecture of radial glia fibers.**

(A-B) Representative images of Cleaved Caspase3 staining (red) in E18.5 mouse brains electroporated with control or EphB6-overexpression plasmids at E15.5. I-VI indicates the sublayers of cortex.

(C-D) Quantitative analysis the ratios of GFP^+^/Cleaved Caspase3^+^ cells in the total population of GFP^+^ cells (C) and the total number of Cleaved Caspase3^+^ cells between the two (D). Bar graphs are plotted as mean ± SEM, n.s., not significant, *p* > 0.05, Student’s *t* test, n = 3 brains. Scale bar, 200 μm in (A-B).

(E-J) Representative images of Nestin immunostaining showing radial glia fibers in E18.5 cortex with overexpression of EphB6 and control plasmid at E15.5. Radial glial fibers are labeled with Nestin antibody (red), and cortical neurons are shown in green. Scale bar, 50 μm.


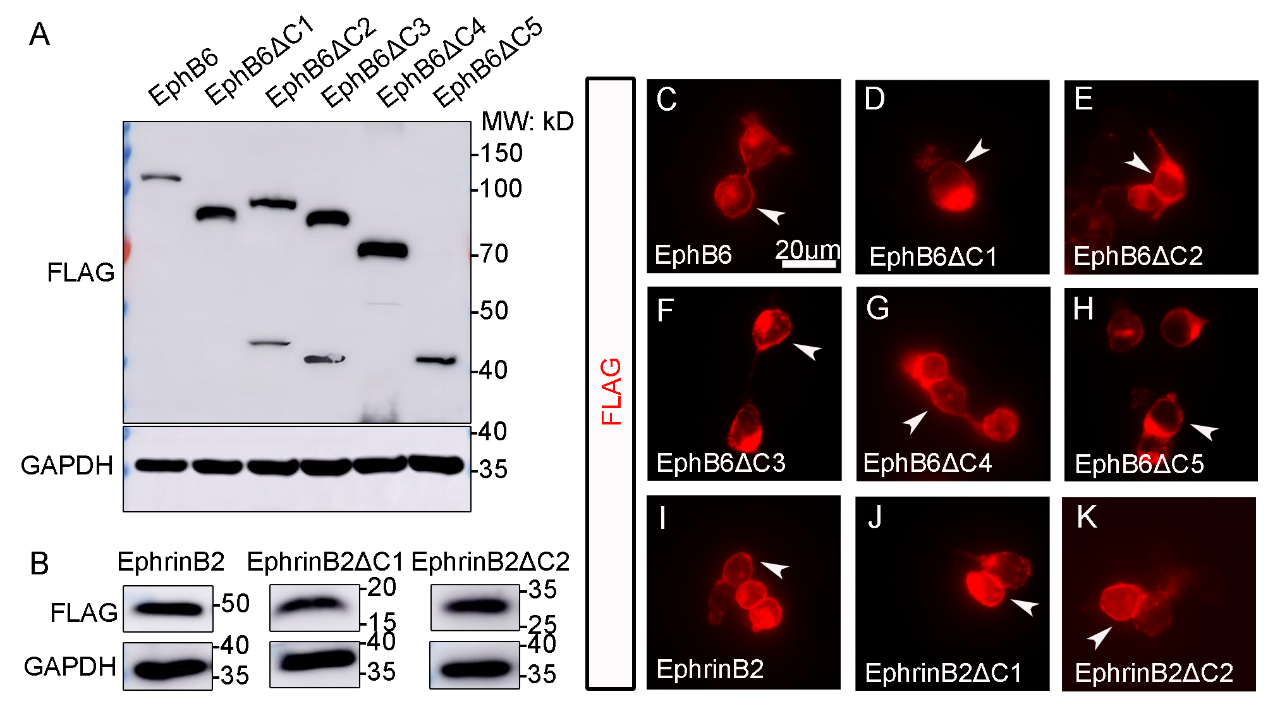
**Fig. S4 Verification of expression of EphB6, EphrinB2 and their truncated forms in HEK293T cells.**

(A) Western blot of HEK293T cells transfected with the indicated plasmids in verifying the expression of full-length and truncated forms of EphB6.

(B) Western blot of HEK293T cells transfected with the indicated plasmids in verifying the expression of full-length and truncated forms of EphrinB2.

(C-K) Immunostaining of FLAG-tagged EphB6, EphrinB2 and their truncated forms shows that they were localized on the cell membrane of HEK293T cells. Scale bar, 20 μm.


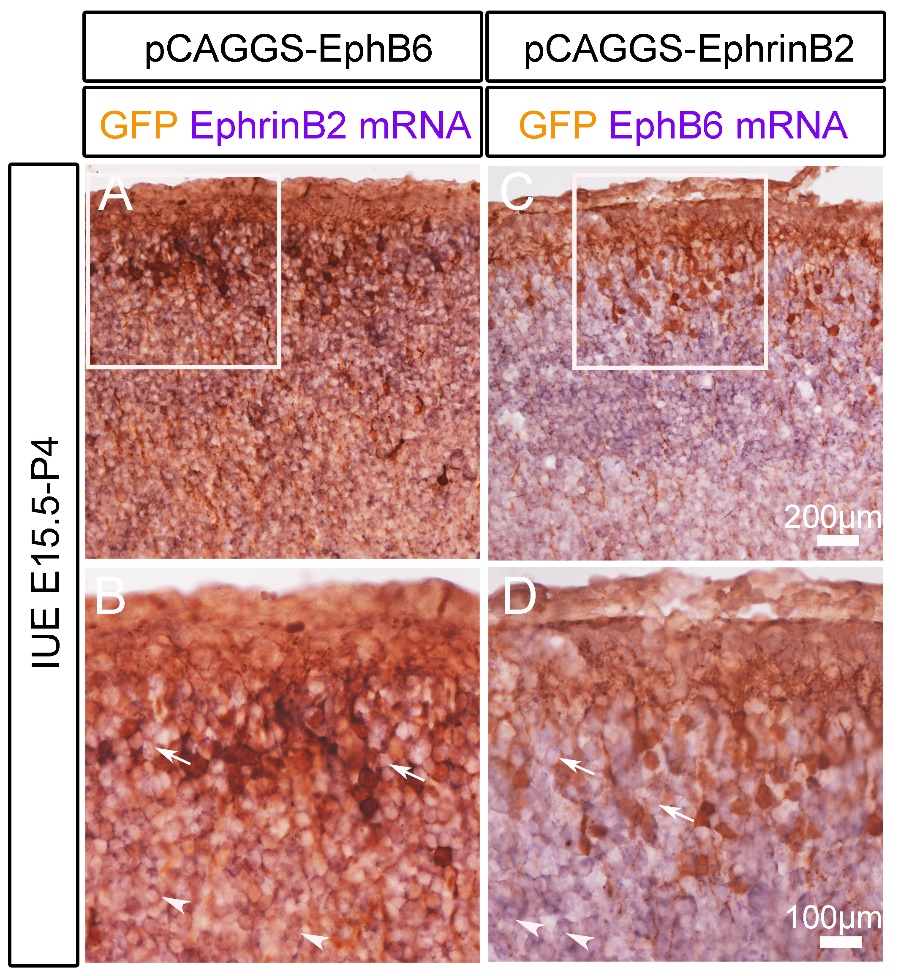
**Fig. S5 Overexpression of EphB6 or EphrinB2 does not induce any changes in the expression of EphrinB2 or EphB6, respectively, in neighboring untransfected neurons.**

(A-B) Double ISH for EphrinB2 mRNA and GFP immunostaining show that EphB6 overexpression had no effect on the expression of EphrinB2 in neighboring neurons (arrows), comparing to neurons far away (arrow heads). Panel B is the enlarged boxed area in panel A.

(C-D) Double ISH for EphB6 mRNA and GFP immunostaining show that EphrinB2 overexpression had no effect on the expression of EphB6 in neighboring neurons (arrows), comparing to neurons far away (arrow heads). Panel D is the enlarged boxed area in panel C.

Scale bars, 200 μm in A and C, 100 μm in B and D.


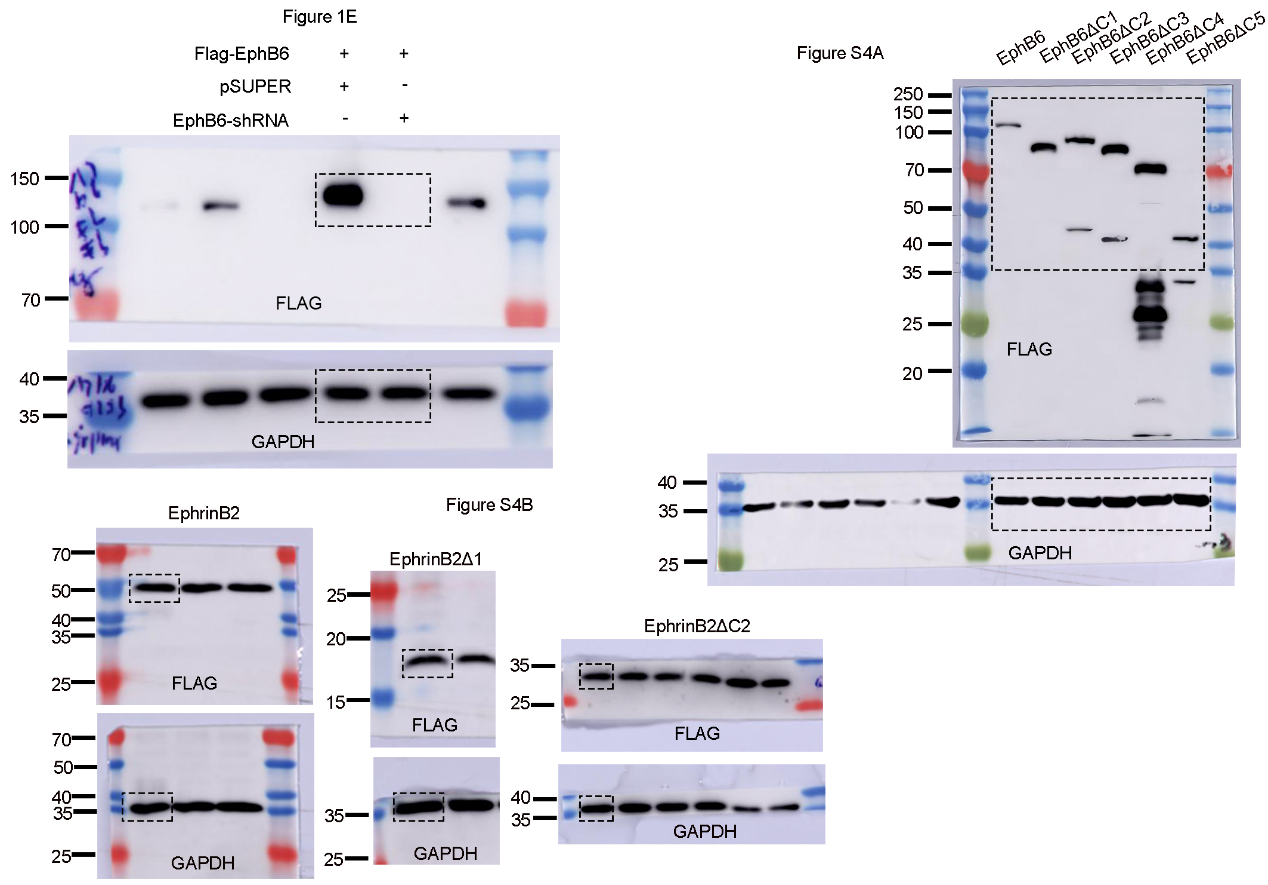
**Fig. S6 Full-length uncropped pictures of the Western blots presented in the figures.**
